# XAV939-Mediated ARTD Activity Inhibition in Human MB Cell Lines

**DOI:** 10.1371/journal.pone.0124149

**Published:** 2015-04-02

**Authors:** Cristiano Renna, Roberta Salaroli, Claudia Cocchi, Giovanna Cenacchi

**Affiliations:** Department of Biomedical and Neuromotor Sciences, Alma Mater Studiorum University of Bologna, Bologna, Italy; University of Parma, ITALY

## Abstract

Diphtheria toxin-like ADP-ribosyltransferases 1 and 5 (ARTD-1, ARTD-5) are poly ADP-ribose enzymes (PARP) involved in non-homologous end-joining (NHEJ), which is the major pathway of double-strand break (DSB) repair. In addition, ARTD-5, or Tankyrase (TNKS), is a positive regulator of the WNT signaling implicated in the development and biological behavior of many neoplasms, such as Medulloblastoma (MB), in which radiotherapy is an essential part of the treatment. The use of radiosensitizing agents may improve the therapeutic index in MB patients by increasing the efficacy of radiotherapy, while reducing toxicity to the neuroaxis. ARTD-5 seems to be a good molecular target for improving the current treatment of MB. In this study, we used the small molecule XAV939, a potent ARTD-5 inhibitor with a slight affinity for ARTD-1, in different human MB cell lines. XAV939 inhibited the WNT pathway and DNA-PKcs in our MB cells, with many biological consequences. The co-administration of XAV939 and ionizing radiations (IR) inhibited MB cells proliferation and clonogenic capacity, decreased their efficacy in repairing DNA damage, and increased IR-induced cell mortality. In conclusion, our *in vitro* data show that XAV939 could be a very promising small molecule in MB treatment, and these results lay the basis for further *in vivo* studies with the aim of improving the current therapy available for MB patients.

## Introduction

ARTDs, a superfamily of 17 proteins, play a crucial role in different cellular functions such as DNA damage detection and repair, chromatin modification, mitotic apparatus formation, and cell death by transferring ADP-ribose unit or units onto specific molecular targets (a post-translational modification process referred to as “PARsylation”). Given this essential role in DNA mechanism repair, several studies have been carried out to explore the therapeutic potential of ARTDs specific inhibitors. Thus, both *in vitro* and mice studies indicate the rationale to combine ARTDs inhibitors with DNA damaging agents in many different tumor types. On October 24, 2014, AstraZeneca announced that the Committee for Medicinal Products for Human Use (CHMP) of the European Medicines Agency (EMA) has adopted a positive opinion recommending the marketing authorization of Lynparza (olaparib, an ARTD-1 and ARTD-2 inhibitor) as monotherapy for the maintenance treatment of patients with relapsed BRCA-mutated high grade serous epithelial ovarian, fallopian tube, or primary peritoneal cancer. [1–6].

The ARTD family member, ARTD-5, otherwise known as tankyrase (TNKS) has been shown to be involved in a multitude of critical cellular processes; it consists of two isoforms (TNKS1 and TNKS2), which share 85% amino acid sequence identity and have overlapping functions [7–10].

TNKS1 regulates DNA repair via PARsylation mediated stabilization of DNA-dependent Protein Kinase catalytic subunit (DNA-PKcs). The depletion of TNKS by siRNA-mediated knockdown or its inhibition resulted in proteasome-mediated DNA-PKcs degradation. The failure of the non-homologous end-joining (NHEJ) function on DNA damage mechanism, the major pathway of DSB repair, is also evident. Correct DNA-PKcs activity is critical for the NHEJ mechanism; hence, TNKS inhibition results in an increased sensitivity to DNA damage agents [11–13].

Furthermore, it was shown that TNKS is a positive regulator of WNT signaling. TNKS-mediated PARsylation of AXIN induces the degradation of AXIN, the concentration-limiting component of the β-catenin destruction complex, and therefore, WNT pathway activation. Thus, TNKS inhibition antagonizes the WNT pathway by promoting Axin stabilization [14].

Alterations of the WNT pathway often occur in Medulloblastoma (MB), a highly invasive embryonal neuroepithelial tumor of the cerebellum (WHO, grade IV) [15–18]. By analyzing gene-expression profiles, a recent study has proposed four subtypes of MB, each of which is characterized by a distinct genetic profile, oncogenic pathway activation, and clinical outcomes. Specifically, MB subgroup A is characterized by the WNT pathway, subgroup B is characterized by SHH signaling, and C and D are characterized by the expression of neuronal differentiation genes [19,20].

Radiotherapy is particularly effective in MB treatment. Ionizing radiations (IR) induce different DNA damage typologies; the most critical lesions are DSBs [13]. Unfortunately, radiotherapy is notorious for causing late-onset side effects, not only regarding the developing cortex and deep brain structures, but also the posterior fossa; the risk is higher in younger patients [21–26].

The use of radiosensitizing agents, which target specific tumor cell characteristics, such as their replication dependency and DNA repair defects, may improve the therapeutic index by increasing the efficacy of radiotherapy, while reducing the toxicity and damage to the developing brain.

This therapeutic strategy could be particularly useful in highly proliferative high-grade childhood brain tumors such as MB, which arise in largely non-replicative normal tissues with proficient DNA repair [27].

In this regard, TNKS seems to be an optimal molecular target to improve the currently available therapy for MB, given its crucial role in the NHEJ pathway and, consequently, in DSB repair. Moreover, as mentioned previously, TNKS depletion results in a WNT pathway inhibition that is strongly involved in MB development and biological behavior.

The small molecule XAV939 [14] is known to have a high affinity for TNKS proteins. Indeed, the administration of this compound in several cell cultures results in TNKS PARP activity inhibition and, therefore, a WNT pathway inhibition (mediated by Axin increasing) and DNA-PKcs protein level reduction. XAV939 also binds to ARTD-1, but with approximately 10-fold lower affinity than to ARTD-5A/ARTD-5b [14,28,29]. Therefore, in this study, we investigated the rationale for combining XAV939 administration with IR in MB using an *in vitro* model system. We used the small molecule XAV939 in two different human MB cell lines, evaluating the radiosensitizing potentiality of this inhibition in this kind of embryonal tumor.

## Materials and Methods

### Cell culture

The study of the therapeutic potential of ARTD-1 and TNKS inhibition was conducted on two different human MB cell lines: ONS-76 (derived from classic MB) and DAOY (desmoplastic MB) [30–31]. (ONS-76 were kindly provided by Dr. Charles G. Eberhart, John Hopkins University, Baltimore, MD, with the agreement of Dr. Mike Bobola, University of Washington, Seattle, WA). DAOY were purchased from American Type Culture Collection (ATCC; Manassas, VA).

ONS-76 and DAOY cell lines were maintained, respectively, in RPMI 1640 (Roswell Park Memorial Institute medium; Euroclone, Milan, IT), supplemented with 10% heat-inactivated fetal bovine serum (FBS; Euroclone, Milan, IT), MEM (Minimum Essential Medium with earle’s salts; Euroclone, Milan, IT), supplemented with 10% heat-inactivated fetal bovine serum (FBS; Euroclone, Milan, IT), and 1% sodium pyruvate (Eurobio, courtaboeuf Cedex, FR). Both media were supplemented with 1% penicillin-streptomycin (Euroclone, Milan, IT), 1% 2 mM L-glutamine (Euroclone, Milan, IT), and 1% non-essential amino acids (Euroclone, Milan, IT). Cell cultures were maintained at 37°C in 5% CO_2_.

### Protein extraction and Western Blots

ONS-76 and DAOY nuclear and total proteins were extracted and Western Blotting (WB) was performed, as previously described [32]. The following primary antibodies were used at the respective dilutions in blocking solution: rabbit polyclonal anti-β-catenin (H-102; Santa Cruz Biotechnology, Santa Cruz, CA, USA) 1:1000; mouse monoclonal anti-DNA-PKcs 1:1000 (1C12; Abcam, Cambridge, UK); goat polyclonal anti-Axin (R20; Santa Cruz Biotechnology, Santa Cruz, CA, USA) 1:500; polyclonal anti-β-actin (I-19; Santa Cruz Biotechnology, Santa Cruz, CA, USA) 1:1000; and mouse monoclonal anti-lamin B1 (ZL-b, ABcam, Cambridge, UK) 1:400. Primary antibodies were detected with horseradish peroxidase (HRP)-labelled secondary antibodies at the respective dilutions in blocking solution: anti-rabbit (GE Healthcare Europe GmbH, Freiburg, DE) 1:1000; anti-mouse (GE Healthcare Europe GmbH; Freiburg, DE) 1:10000; and anti-goat (Santa Cruz Biotechnology, Santa Cruz, CA) 1:1000. Total DNA-PKcs, β-actin, and Axin were detected on total protein extracts, while β-catenin and lamin B detection was performed on nuclear extracts. β-actin and lamin B were used to check for total and nuclear loading, respectively. The experiments were performed at least three times.

The electrophoretic separation for the detection of the high molecular weight DNA-PKcs (460 kDa) and of the normalizer β-actin, was performed by using a 3.5%–12% gradient SDS-PAGE: acrylamide 30% plus 0.8% bisacrylamide cross-linker in 1.5 mol/L Tris/HCl buffer, pH 8.8, containing 0.05% sodium dodecyl sulfate (SDS). BioRad Protean II (BioRad, Hercules, CA, USA) was used to cast two 16 cm gels, 1.5 mm thick. A peristaltic pump and a gradient mixer (Sigma Aldrich Corporation, St. Louis, MO, USA) were also used to cast a resolving gel. After the resolving gel had set (approximately 3 h), the stacking gel, consisting of 3% acrylamide in 0.125 mol/L Tris/HCl, pH 6.8, and 0.1% SDS, was poured above the resolving gel and allowed to polymerize around the comb that was inserted to form the sample lines (10 X 1 cm). The samples were resuspended in Laemli Buffer (2% SDS, 10% glycerol, 5% 2-mercaptoethanol, 0.002% bromphenol blue, 0.125 M Tris HCl, pH ~6.8), while the gels were setting, and 25 μl were applied to each lane. The gels were run at 21 mA overnight in a tank buffer containing 1.44% glycine, 0.3% Tris, and 0.1% SDS, pH 8.8, using a thermocirculator set at 0°C. The next day, the gels were blotted with a limiting current of 1A for 5 h onto nitrocellulose using a transfer buffer that contained 1.44% glycine, 0.3% Tris, pH 8.3, and 20% methanol. The BioRad blotting tank (BioRad, Hercules, CA, USA) contained plate electrodes and a supercooling coil. The equipment was attached to a thermocirculator set at -13.5°C. Nitrocellulose membranes were incubated with anti-DNA-PKcs and anti-β-actin antibody.

### XAV939 and IR treatment

DAOY and ONS-76 cell lines were plated 24 h prior to treatment with 5 μM of XAV939 or with equal DMSO volume, with radiations or with a co-administration of drug and radiations (cells were treated with the small molecule and irradiated 8 h later).

XAV939 (Sigma-Aldrich Corporation, St. Louis, MO, USA) was solubilized in DMSO to a stock concentration of 50 mM, which was diluted to a working concentration of 5 μM. Cultures were maintained under these conditions for the duration of the designated time course. Controls were exposed to DMSO alone.

Cells were irradiated with an Irradiateur Biologique 437C (CIS-BIO; Cedex, France) γ-ray machine at a dose of 2 or 10 Gy and a dose rate of approximately 2 Gy/min.

Post treatments, the cells were analyzed at different time points. Cell growth, colony formation, mortality rate, and DNA repair efficacy were evaluated.

### Cell growth analysis

ONS-76 and DAOY MB cell lines were plated at a density of 200,000 and 500,000 viable cells per T25 flask, respectively, and treated as described above.

Cell number and viability were assessed using the Trypan blue reagent (Sigma-Aldrich Corporation, St. Louis, MO, USA). The cells were collected by trypsin-EDTA treatment, pelleted by gentle centrifugation, and resuspended in Trypan blue reagent to count viable cells using a light microscope every day for 4 days after treatments. Multiple replicate flasks were assayed at each time point in each experiment, and each sample was examined in three independent experiments. At each time point, we report the mean cell number from all the scored flasks; error bars display SE.

### Cell mortality assay

In order to evaluate cell mortality, 200,000 cells were seeded in T25 culture flasks and collected 48, 72, and 96 h after treatments. Irradiated and control samples from each line were harvested at corresponding time-points, trypsinized, and total cells were suspended 1:1 in Trypan Blue solution. Dead and total cells from every sample were counted using a Neubauer chamber. Multiple replicate flasks were assayed at every time point in each experiment, and each sample was examined in three independent experiments. At each time point, we report the mean cell number from all the scored flasks; error bars display SE.

In a second set of experiments, cells were treated with XAV939 at the indicated concentration and irradiated with a double 2 Gy dose. Mortality rate was then calculated.

In the histograms, cell mortality of the treated cells was normalized to DMSO control cells.

### Clonogenic assay

ONS-76 and DAOY cell lines were seeded in low serum growth medium (1% FBS) at 300 and 500 cells/well, respectively, into 6-well plates. Sixteen hours after plating, cells were treated as described above. Media were replenished every three days until colony formation was observed. Colonies were stained with a solution of 2 mg/mL crystal violet in buffered formalin and observed under a light microscope. Each assay was made at least three times and only colonies of more than 50 cells were counted.

The survival fraction at 2 Gy (SF_2_), determined as the ratio of the number of colonies formed by irradiated cells to the number of colonies formed by non-irradiated cells, was also calculated.

### Neutral Comet Assay

In order to avoid high lyses temperatures, which could alter the assay reliability, we used the protocol described by Olive and Banàth, with some modifications [33, 34]. MB cell lines treated and untreated with XAV939 (5μM) were exposed to 10 Gy. Neutral Comet Assay was performed 0, 16, and 24 h after radiation. Non-irradiated cells were used as controls. In short, after treatments cells were resuspended in low melting agarose and immediately placed on microscopic slides (pre-coated with 0.5% normal melting agarose) under coverslips and, after agarose solidification, they were lysed with specific basic buffer (2% sarkosyl, 0.5M Na_2_EDTA, pH 8.0, stored at 4°C) for at least 4 h at 37°C. After lysis, the slides were washed in electrophoresis buffer (90 mM Tris buffer, 90 mM boric acid, 2 mM Na_2_EDTA, pH 8.5) and placed in a horizontal gel electrophoresis chamber filled with fresh electrophoresis buffer. Electrophoresis was conducted at 7 mA (0.6 V/cm) for 25 min and at a temperature not higher than 15°C. The slides were then washed in distillated water, stained with a 20 μg/mL ethidium bromide solution for 20 min, and covered with cover glasses. The image analysis was performed on pictures of 50 randomly selected comets per slide for each sample. Experiments were repeated at least three times. Data show mean comet TM and SD.

Pictures were captured using a fluorescence microscope at 50X magnification. Free software, CASP, was used to measure the Tail Moment (TM, % Tail DNA x length Tail) and quantify DNA damage. Cells treated with hydrogen peroxide (at 10 μM for 15 min) were used as inner controls to verify the specificity of the method used to recognize mainly DSBs [34].

### Statistical analysis

Data are expressed as means ± SD. Statistical analyses were performed using Dunnett’s test (WB densitometry analyses), one-way ANOVA, and Bonferroni’s post-test (clonogenic forming assay, cell mortality) or two-way ANOVA test and Bonferroni’s post-test (growth curves assay), and employed Prism 5.0 software (GraphPad Software, La Jolla, CA). Statistical significance was set at *p≤*.05.

## Results

### XAV939 inhibits WNT pathway and DNA-PKcs in MB cell lines

**Fig 1:**
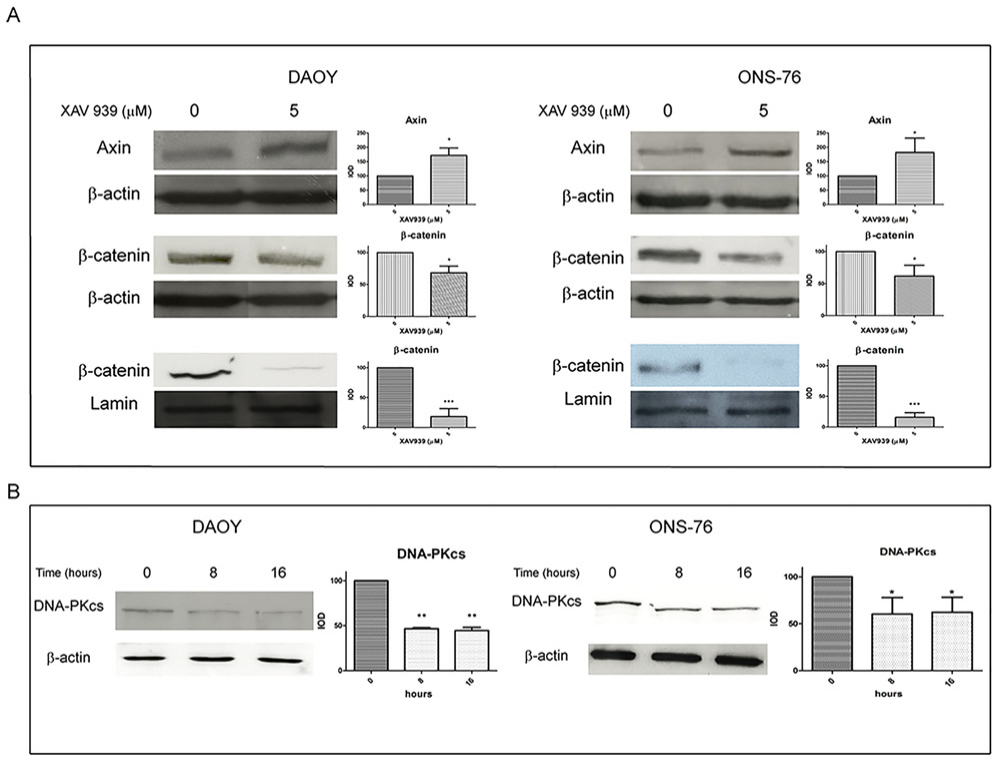
XAV939 inhibits TNKS PARP-activity in MB cell lines. WB analysis and densitometry of total and nuclear MB cells extracts after XAV939 treatment: MB cell lines (DAOY, ONS-76) were treated with 5 μM XAV939 or with an equal volume of DMSO. **A.** Both cell lines showed an increase in total Axin protein levels at 8 h after treatment (80% and 70% respectively in ONS-76 and DAOY compared to DMSO treated control, *p* <. 05), followed by a β-catenin decrease in total (30%, *p* <. 05) and, in particular, in nuclear extracts, at 16 h after drug administration (80% reduction compared with control, *p* <. 001). **B.** XAV939 induced a DNA-PKcs protein level reduction of about 40% at 8 h and 16 h after treatment compared to non-treated control cells (*p* <. 05 in A, *p* <. 01 in B). Densitometry data (mean ± s.e.) were normalized with β-actin (for total extracts) and lamin-b (for nuclear extracts) and are representative of the results derived from three independent experiments.

XAV939 is known to induce WNT signaling inhibition by increasing Axin protein levels, thereby promoting β-catenin degradation [14]. We found an effective increase in total Axin levels, 8 h after treatment with XAV939 (5 μM) and a decrease in β-catenin protein levels, both in total and in nuclear extracts, at 16 h. Densitometry analyses revealed a 70% increase of total Axin protein levels in DAOY cells and an 80% increase in ONS-76 cells, treated with XAV939 (5 μM) compared to DMSO treated control cells. Total β-catenin protein levels decreased by about 30% in both cell lines compared to controls; whereas, in nuclear fraction, we found an 80% reduction of β-catenin levels (Fig 1A).

Moreover, since TNKS PARP activity is critical for DNA-PK catalytic subunit (DNA-PKcs) stability [11], we tested whether XAV939 treatment affected DNA-PKcs protein levels in our cell lines. Examination of DNA-PKcs levels after 8 h and 16 h of XAV939 treatment revealed a significant protein abundance decrease compared to controls: we found a 50% reduction in DAOY cells and a 40% reduction in ONS-76 cells (Fig 1B).

The increase in Axin protein levels, followed by β-catenin reduction, and DNA-PKcs levels inhibition confirmed the efficacy of XAV939 in affecting TNKS PARP activity on these MB cell lines.

### The co-administration of XAV939 and IR inhibits MB cell proliferation and clonogenic capacity

XAV939 and IR effects on the viability of MB cell lines were assessed by comparing growth curves. Cells were treated with 5 μM of XAV939 or with an equal DMSO volume, with radiation (γ-ray, dose 2 Gy, dose rate ~ 2 Gy/min) or with a co-administration of drug and radiation.

**Fig 2:**
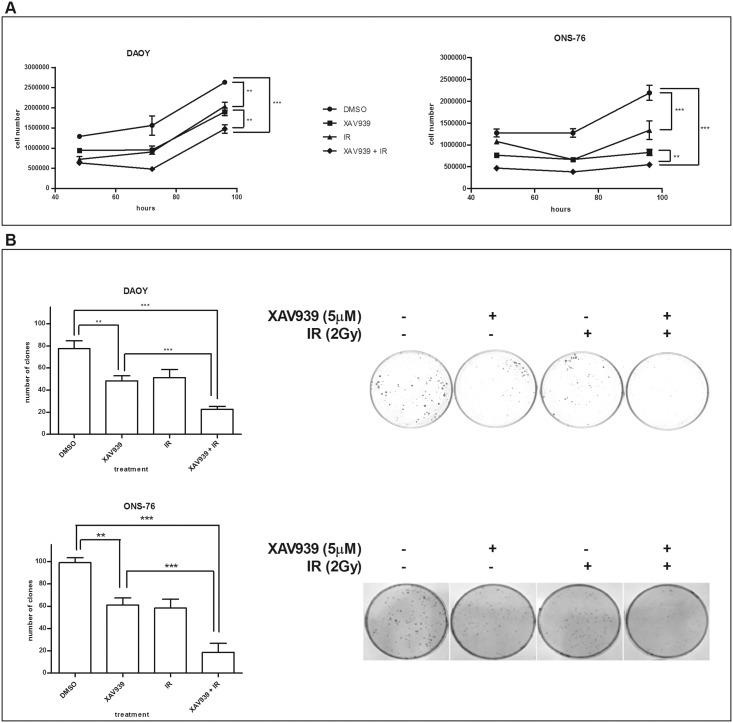
XAV939 impairs the clonogenic and proliferative capacity and enhances MB cells radio-sensitivity. **A.** *Growth curves assay*. XAV939 (5 μM) treated cells and IR (2 Gy) treated cells showed a similar growth rate with about 40% (DAOY) and 30% (ONS-76) reductions compared to DMSO control cells (*p* <. 01). In both cell lines, the co-administration of XAV939 and IR induces a massive cell proliferation inhibition with a decrease of about 70% (*p* <. 001). Each point represents the mean ± s.e. of three independent assays. **B.** *Clonogenic forming assay*. XAV939 alone (5 μM) inhibits a clone-forming ability in both MB cell lines as well as IR treatment (2 Gy), with a clonogenic capability reduction of about 38% (*p* <. 01). The IR and drug co-administration induces a drastic inhibition of clone-forming in both cell lines (70% reduction in DAOY cells and 81% in ONS-76 compared with control cells, *p* <. 001).

Fig 2A shows that the co-administration of XAV939 and IR strongly suppresses MB cell proliferation in both cell lines. In DAOY cells, the greatest proliferation inhibition was observed 72 h post IR in XAV939 pre-treated cells, with a 69.2% reduction in cell proliferation compared to DMSO control cells (*p* <. 001, two-way ANOVA) and a 42.65% decrease in cell number relative to DMSO irradiated cells (*p* <. 01, two-way ANOVA). In ONS-76 cells, we found a massive inhibition in XAV939 and irradiated cells at 96 h after IR. At this time point, we detected a 75% cell number reduction compared to DMSO control cells (*p* <. 001, two-way ANOVA) and a 34% reduction compared with DMSO irradiated cells (*p* <. 001, two-way ANOVA). Furthermore, our data showed that XAV939 alone is able to inhibit MB cell growth: the greater proliferation decrease observed was at 72 h after drug administration (38.9% reduction in DAOY cells and 48.1% in ONS-76 cells, *p* <. 01, two-way ANOVA).

Analogous results were found for clonogenic capability. Both MB cell lines showed a significant reduction (38%) of the colony formation after exposure to 5 μM XAV939 (*p* <. 01, one-way ANOVA). Nevertheless, XAV939 and IR treated cells showed the greatest inhibition of clonogenic formation compared to the other samples. In DAOY cells, we observed a 70.6% clone number reduction in XAV939 and irradiated cells compared to control cells (*p* <. 001, one-way ANOVA) and 55.6% relative to DMSO irradiated cells (*p* <. 01, one-way ANOVA); whereas, in ONS-76 cells, reductions of 81% (*p* <. 001, one-way ANOVA) and 67.9% (*p* <. 05, one-way ANOVA) were observed, respectively (Fig 2B).

By using clone formation data, we calculated SF_2_. We found that XAV939 reduced the SF_2_ from 0.66 to 0.29 in DAOY cells and from 0.58 to 0.19 in ONS-76 cells, indicating that PARP activity inhibition halved the percentage of cells that survive radiation treatments. This confirms a concrete enhanced radiosensitivity in these MB cells.

### XAV939 affects efficacy of DNA repair after IR

The critical cellular lesions in relation to cytotoxic effects generated by IR are DNA-DSBs [12,13]. The DNA-PK pathway is one of the two most important pathways involved in DNA-DSBs repair [1]. As TNKS is a positive regulator of this pathway by inducing DNA-PKcs stability [10], we investigated whether XAV939 affects DNA repair efficacy after IR treatment. On the other hand, ARTD-1 inhibition by XAV939 could also have a critical role in NHEJ mechanisms [1].

**Fig 3:**
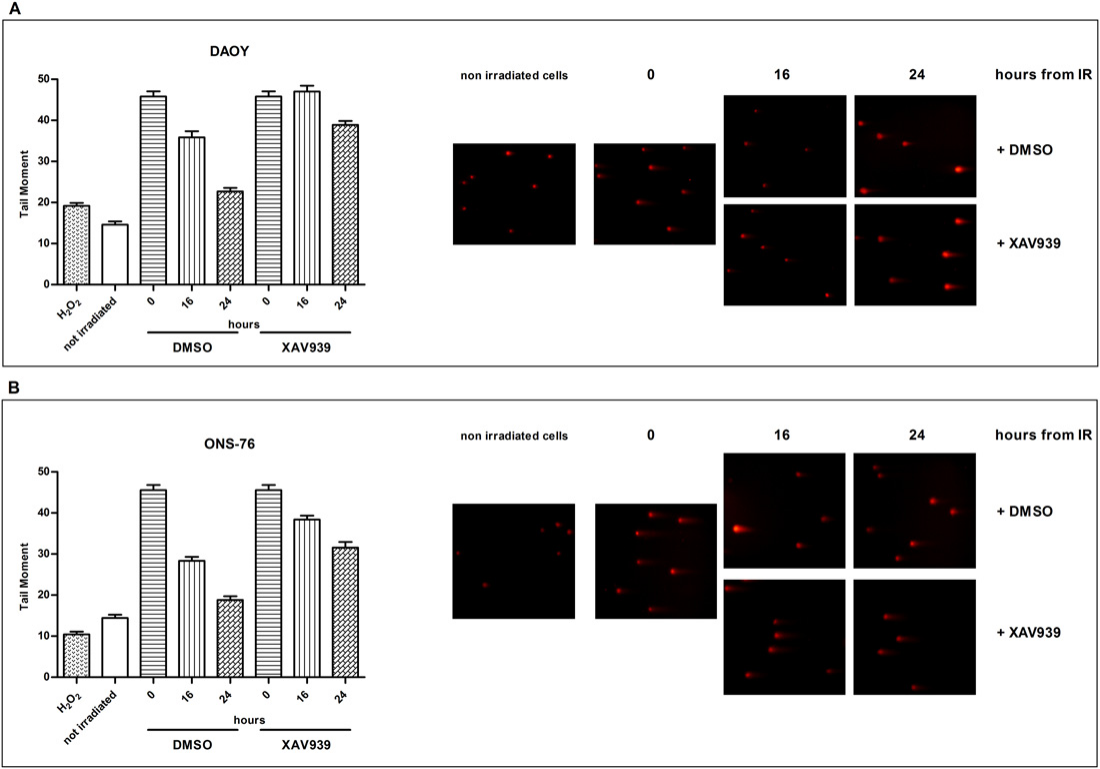
XAV939 affects the DNA repair efficacy of MB cell lines. Neutral Comet Assay was performed on IR treated cells (10 Gy), with or without XAV939 administration, at different time points post radiations (0, 16, and 24 h). Histograms represent mean ± s.e. of TM measured in at least three independent experiments for each treatment and time point. The greatest TM was observed immediately after IR treatment in both cell lines (A: DAOY, B: ONS-76). XAV939 induces enhanced TM values at 16 h and 24 h after IR compared to DMSO irradiated cells indicating a minor DNA repair capacity. On the right of the figure, a representative example of comets for each treatment obtained in DAOY (upper) and ONS-76 (down) cells.

The neutral comet assay was performed for this purpose, because of its ability to highlight predominantly DSBs [33,34] (Fig 3). As a measure of DNA damage, we used TM, the most common parameter, for analyzing the comet assay results. We utilized hydrogen peroxide (at 10 μM for 15 min) as a negative control, since it induces principally SSBs; whereas, DSBs are considered very rare events [34].

First, we assessed the repair kinetics of IR-induced DSBs in DAOY and ONS-76 cells analyzing TM at various time points after IR (0, 16, and 24 h, 10 Gy dose). Fig 3A and 3B show that the highest TM value was observed, as expected, immediately after IR in both cell lines. At 16 h after irradiation, we observed a decrease in TM, whereas 24 h after IR, we detected a TM value similar to that of non-irradiated cells, suggesting that DNA damage was repaired. Moreover, hydrogen peroxide did not induce a significant increase in TM compared to non-irradiated cells, confirming the effectiveness of the method in highlighting mainly DSBs.

XAV939 and IR treated cells (both in DAOY and ONS-76 cells) showed an evident delay in their capability to repair DNA damage caused by radiation. The repair kinetics of these cells are slower than DMSO irradiated cells. Indeed, in XAV939 and IR treated cells at the same time points, we consistently observed a much higher TM compared to IR treated cells. In DAOY cells, 16 h after the radiations, we observed a TM comparable to the TM value found immediately after IR and, at 24 h, XAV939 pre-treated cells showed a double TM value compared to DMSO control cells. In ONS-76 cells, at 24 h after IR, we detected an almost double TM in XAV939 pre-treated cells in relation to DMSO control cells; whereas, it was higher than 30% at 16 h. These results suggest a poor efficacy of DNA damage repair in XAV939 and IR treated cells compared to controls.

### XAV939 increases IR-induced cell mortality

We evaluated, by trypan blue assay, whether cell proliferation inhibition and clonogenic formation reduction correlated with an increase in IR-induced cell mortality.

**Fig 4:**
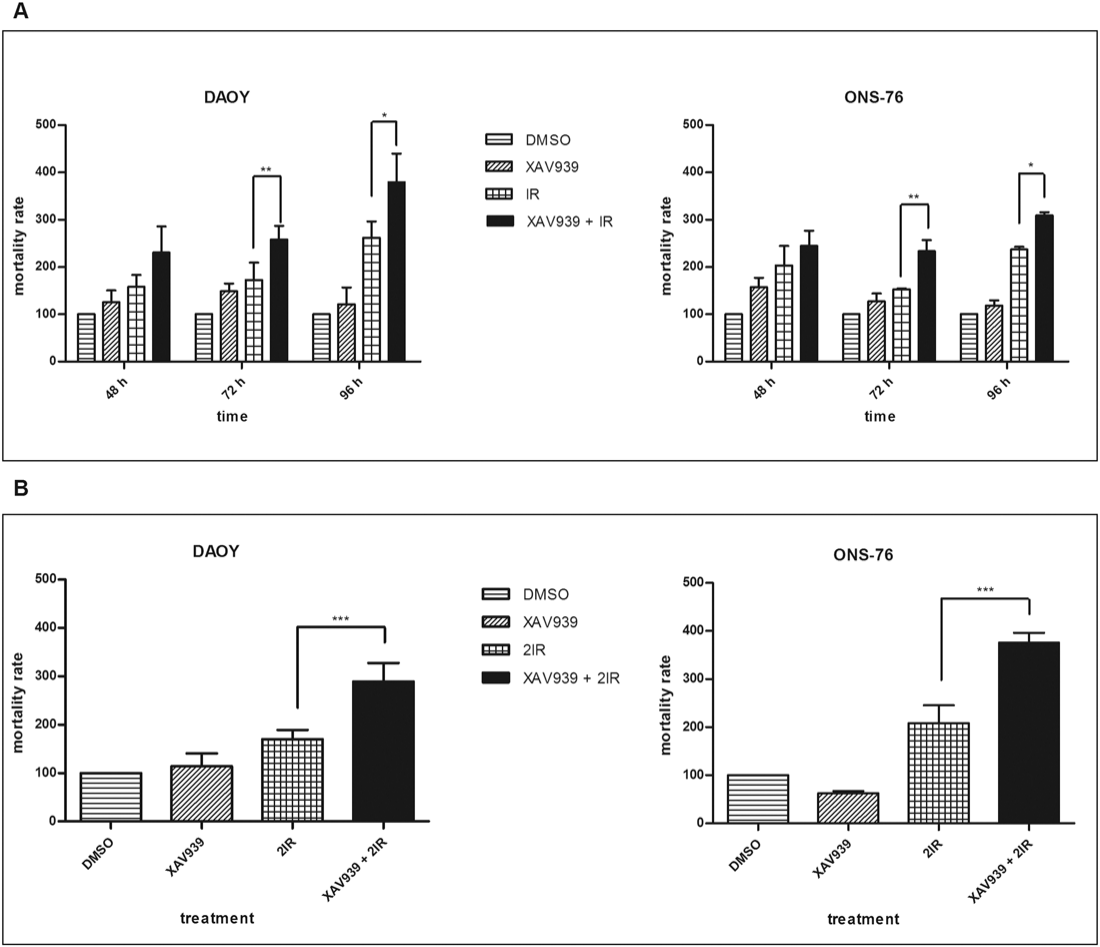
XAV939 improves cell mortality IR-induced. Histograms represent means ± s.e. of three independent experiments. Mortality rates of XAV939 treated cells, IR treated cells, XAV939, and IR treated cells are reported as relative percentages compared to DMSO control cells. **A.** Cell mortality after a 2 Gy dose administration: XAV939 alone does not induce a significant cell mortality increase in each time point considered. The greatest cell death was observed in XAV939 and IR treated cells in both cell lines, with a statistically significant difference at 72 h and 96 h compared with IR treated cells (*p* <. 01 and *p* <. 05, respectively). **B.** Mortality rate in MB cell lines after two treatments with 2 Gy dose of IR. In both cell lines, cells treated with XAV939 and irradiated, as described, showed a double mortality rate compared to DMSO irradiated cells (*p* <. 001).

DAOY cells irradiated with a 2 Gy dose showed a 16.81%, 10.9%, and 9.3% mortality rate at 48, 72, and 96 h after radiation, respectively. Cells treated with 5 μM of XAV939 before IR showed 23.5%, 17%, and 13% mortality rate with a 6.77%, 6.1%, and 3.7% increase (data not shown). Normalizing data to controls, we observed, at 72 h and 96 h after radiation, about a 40% and a 45% increase in cell mortality in cells pre-treated with XAV939 compared to only irradiated ones (Fig 4A): this difference was considered statistically significant (*p* <. 01 and *p* <. 05, respectively, one-way ANOVA). ONS-76 cells, at the same time points, showed a 10.61%, 9.11%, and 10.43% mortality rate after IR. In this cell line, XAV939 induced a slight increase in IR-mediated cell mortality: 12.96%, 13.49%, and 13.23% with a difference of 2.35%, 4.38%, and 2.8%, respectively (data not shown). When we normalized data to controls, a statistically significant increase in cell mortality between IR treated cells, XAV939, and irradiated cells was observed at 72 h (52.8%, *p* <. 01 one-way ANOVA) and at 96 h (30%, *p* <. 05, one-way ANOVA).

Despite these results, we did not observe as strong an increase in cell mortality as expected. Testing the hypothesis that a single 2 Gy dose was enough to affect proliferation and clonogenic capability, but not enough to induce a massive increase in cell mortality, we irradiated with a second 2 Gy dose. DAOY cells treated with XAV939 and γ -rays, with two doses of 2 Gy, showed a 30.95% mortality rate compared to 16.17% of cells not treated with the small molecule but only irradiated two times (*p* <. 001, one-way ANOVA). Similarly, in ONS-76 cells, XAV939 administration induced an almost double mortality rate in cells irradiated, as described, compared to cells treated with IR alone (17.75% and 9.8%, respectively, *p* <. 001, one-way ANOVA). Fig 4B shows data normalized to non-irradiated controls: we report, in both cell lines, about a 100% increase in mortality rate of XAV939 and irradiated cells compared to cells treated with IR alone, as described. These data confirm an enhanced MB cell radiosensitivity mediated by XAV939 administration. Finally, a statistically significant difference in mortality rate was not detected between XAV939 treated cells and DMSO control cells, in both cell lines and at all time points considered.

## Discussion

In recent years, increasing interest has been focused on ARTD proteins because of their strong involvement in the DNA repair mechanism. ARTD inhibitors were first developed as sensitizers to DNA-damaging chemotherapy or IR, and several highly potent and specific ones have been used in clinical trials in cancer patients [2–4]. Olaparib (AZD2281) is an oral PARP-1 and PARP-2 inhibitor that has been commercialized by Astra Zeneca [6]. Nonetheless, Dregalla and co-workers focused on the therapeutic potential of ARTD-5 inhibition due to its crucial role in the NHEJ pathway by using XAV939 small molecule [11]. On the other hand, TNKS, but not PARP-1/PARP-2, is implicated in the WNT pathway by regulating Axin protein levels [14]. Recent *in vitro* and *in vivo* studies showed that WNT pathway inhibition suppresses MB growth, promoting cell cycle arrest followed by apoptosis [35,36].

In our *in vitro* study, XAV939-mediated ARTD-1 and ARTD-5 inhibition impairs the growth and clonogenic capability of MB cell lines, but does not significantly increase cell mortality, indicating the low toxicity of the drug. The most notable results observed on cell proliferation occurred following the co-administration of XAV939 and IR. In these experiments, we treated cells with a 2 Gy dose that corresponded to the daily fraction of the total radiation dose (36 Gy) usually used in standard MB therapy. At this low dose, we found a massive cell proliferation inhibition in XAV939 pre-treated cells compared to DMSO irradiated cells. The assessment of cell proliferation after irradiation is considered a valid assay for defining cell radiation sensitivity because it provides information on the proliferative capacities of surviving cells after radiation [37,38]. Our data suggest that XAV939 treatment induces an increase in radiosensitivity of MB cells.

Comparable results were found when analyzing the clonogenic capacity of MB cell lines after drug and IR treatments. Indeed, the same trend, obtained by growth curves analyses, was observed using the clonogenic assay, the gold standard for the determination of radiation sensitivity [37,38]. MB cells treated with a combination of XAV939 and γ-rays showed greater clone-forming capability inhibition compared with cells treated with drug or IR alone. The reduction of SF_2_ in both MB cell lines confirmed greater radiosensitivity in XAV939 treated cells.

Given the role of ARTD-1 and TNKS in DNA damage repair and NHEJ, we hypothesized that the observed radiosensitivity was caused by a minor efficacy in DSB mechanism repair. To verify our idea, we used the comet assay, since it is a sensitive, rapid, and simple method for displaying and quantifying cellular DNA damage [33,34]. Under neutral conditions, the assay is able to detect predominately DSBs [32,37]. Nonetheless, the neutral version of the method needs adapting for individual cell types in order to achieve optimal sensitivity [34]. In our experiments, we used the radiation dose of 10 Gy, which is considered an adequate dose for studying DSB repair by the neutral comet assay [34]. In this case, our specific interest was to confirm and quantify whether and how much XAV939 affects DNA mechanism repair in MB cell lines.

The observed kinetic repair trend in control tests was in accordance with results of previous studies [37–48]. Moreover, it has been shown that radiation-induced DSBs are rejoined no later than 20 h in mammalian cell lines [46,48]. In our experiments, both MB cell lines showed, 24 h after IR, a TM comparable to non-irradiated cells, indicating a well-functioning DNA repair mechanism.

Conversely, XAV939 and IR treated cells showed an evident delay in their capability to restore DNA. We focused on 16 h and 24 h time points because it was suggested that damage measured after 4 h or more by treatments is an excellent index of the cell DNA injury restoration ability [48].

Thus, the higher TM value and, therefore, conspicuous DNA damage in XAV939 and IR treated cells at the time points considered, compared to the irradiated control cells, strongly supports the hypothesis that MB cells after XAV939 administration, at the given dose, have limited efficacy to repair DSBs. These data are in accordance with the cell growth and colony-forming ability inhibition previously shown.

In conclusion, XAV939 affected the DNA injury restoration ability, ultimately implying an improvement in the radiosensitivity of MB cells. XAV939-mediated PARP activity inhibition impairs growth and clonogenic capability and increases the mortality rate of MB cell lines. These results are most likely a consequence of TNKS PARP activity inhibition that leads to the WNT pathway inhibition and to the DNA-PKcs instability, causing cell growth reduction and radiosensibility. Since XAV939 also binds to ARTD-1 (also involved in DSB mechanism), but with approximately 10-fold lower affinity than to ARTD-5A/ARTD-5b [14–16], we cannot exclude that part of the shown radiosensitivity is due to this binding; further studies will shed light on this.

Taken together, our *in vitro* data suggest XAV939 as a potential molecule for improving the current therapy for MB. Our results lay the basis for further *in vivo* studies with the aim of designing clinical trials combining XAV939 (or other drugs with similar or better affinity for TNKS) with conventional radiotherapy. Potentially, this will lead to a more effective therapy, with the possibility of reducing radiation doses and, at last, long term side effects.

To date, only *in vitro* studies on XAV939 have been performed; therefore, a study focusing on systemic drug administration is needed to investigate the concrete clinical relevance of this small molecule [49].
